# Cigarette smoke promotes HIV infection of primary bronchial epithelium and additively suppresses CFTR function

**DOI:** 10.1038/s41598-018-26095-z

**Published:** 2018-05-22

**Authors:** S. Chinnapaiyan, R. Dutta, J. Bala, T. Parira, M. Agudelo, M. Nair, H. J. Unwalla

**Affiliations:** Department of Immunology, Institute of Neuroimmune Pharmacology, Herbert Wertheim College of Medicine, Florida International University, Miami, FL USA

## Abstract

Recurrent lung infections are a common cause of morbidity and mortality in people living with HIV and this is exacerbated in smokers even when administered combination antiretroviral therapy (cART). The incidence of pneumonia is increased with smoking and treatment interruption and is directly dependent on viral load in patients when adjusted for CD4 counts. CFTR dysfunction plays an important role in aberrant airway innate immunity as it is pivotal in regulating mucociliary clearance (MCC) rates and other antibacterial mechanisms of the airway. In our earlier work, we have demonstrated that bronchial epithelium expresses canonical HIV receptors CD4, CCR5 and CXCR4 and can be infected with HIV. HIV Tat suppresses CFTR mRNA and function via TGF-β signaling. In the present study, we demonstrate that cigarette smoke (CS) potentiates HIV infection of bronchial epithelial cells by upregulating CD4 and CCR5 expression. HIV and CS individually and additively suppress CFTR biogenesis and function, possibly explaining the increased incidence of lung infections in HIV patients and its exacerbation in HIV smokers.

## Introduction

The advent of combination antiretroviral therapy (cART) has led to a dramatic decline in morbidity and mortality from human immunodeficiency virus (HIV)/AIDS. However, HIV infection is associated with a greater incidence of pulmonary diseases normally associated with aging and their presentation at younger ages. Bacterial pneumonia is the most common cause of hospitalization and intensive care unit admissions in people living with HIV^1^. HIV infection was independently associated with a significantly higher risk for lung infections^2^. Moreover, the incidence of pneumonia is increased with smoking and is directly dependent on viral load in patients when adjusted for CD4 counts^3,4^, suggesting that viral replication also plays an important role in lung infections. In our earlier work, we have demonstrated that primary normal human bronchial epithelial (NHBE) cells express canonical HIV receptors and can be infected with HIV. HIV infection of NHBE suppresses components of the mucociliary clearance (MCC) apparatus. Specifically, we observed that, HIV Tat suppresses cystic fibrosis transmembrane conductance regulator (CFTR) biogenesis and function, infection of differentiated bronchial epithelium disrupts integrity of the epithelial barrier and infection of differentiating NHBE grown at the Air liquid interface (ALI) suppresses ciliogenesis^5^.

CFTR plays a pivotal role in airway innate immunity specifically in modulating MCC. MCC is a primary innate defense mechanism of airways that protects the host from the noxious effects of airborne pathogens, pollutants and allergens^6^. The bacterial spectrum responsible for HIV pneumonias is very similar to that observed in smokers, chronic obstructive pulmonary disease (COPD) patients and in cystic fibrosis^7–10^. Acquired CFTR dysfunction (and MCC dysfunction) plays an important role in the early pathogenesis of these chronic airway diseases in smokers and COPD patients.

Both cigarette smoke (CS) and HIV Tat mediate its suppressive effects on CFTR via a common pathway involving transforming growth factor-beta (TGF-β) signaling^5,11^. CS and TGF-β has been shown to upregulate HIV receptors^12–14^ in different cell types. CS has also been shown to directly enhance HIV replication by reactive oxygen species mediated activation of CYP enzymes^15,16^. Hence CS can enhance HIV replication thereby increasing HIV Tat levels and in turn leading to a synergistic CFTR suppression. This is significant since 60% of people living with HIV also smoke tobacco^17^. We have shown that CS enhances infection of NHBE ALI cultures by R5-tropic virus. We will also show that a combination of CS and HIV infection leads to an additive suppression of CFTR mRNA and a near complete suppression of CFTR function. The implications of these observations on airway innate immunity will be discussed.

## Results

### CS enhances infection of NHBE ALI cultures by R5-tropic HIV strains but not by X4-tropic HIV strains

**Figure 1:**
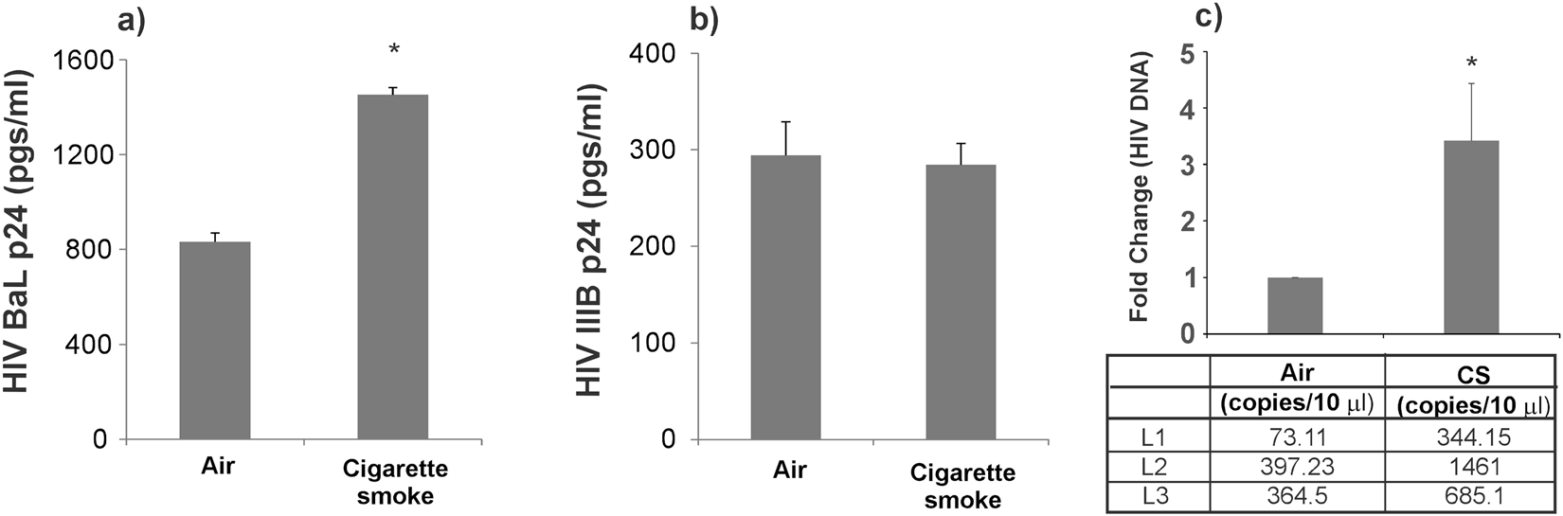
CS enhances replication of R5-tropic HIV. NHBE ALI cultures were exposed to CS prior to infecting apically and basolaterally with 5 ng p24 equivalent of either R5-tropic (HIV BaL) or X4-tropic (HIV IIIB) virus. After 16 hours cells were washed apically and basolaterally with PBS four times to remove any residual input virus. The fourth wash was collected for p24 analysis and measured as day 0 to confirm that all input virus had been removed. On day 3, culture supernatant was collected and analyzed for p24. HIV increases infection of NHBE ALI cultures by the R5-tropic HIV BaL strain (panel a) but not by X4-tropic IIIB strain (panel b). NHBE ALI cultures were infected with single cycle R5-tropic RGH virus. Cells were washed four times with PBS and infection was allowed to proceed. Three days post-infection, experiments were terminated and total DNA was quantitated by qPCR as an index of viral entry. CS increases HIV entry in NHBE ALI cultures (panel c). n = 3 different lungs *significant (p < 0.05). L = Lung number.

In our earlier report, we have shown that differentiated NHBE cells expresses CD4, CXCR4 and CCR5 receptors and can be infected with X4- or R5-tropic virus^5^. Given reports of enhanced HIV loads in smokers^18,19^, we tried to determine if CS increases HIV infection of NHBE cells. Age and lung matched NHBE cultures redifferentiated at the ALI were exposed to CS and then infected with 5 ng of HIV BaL (R5-tropic) or HIV IIIB strain (X4-tropic) HIV as described by us earlier^5^. 16 hours post-infection, cells were washed with phosphate buffer saline (PBS) four times. The fourth wash was collected for p24 analysis and measured as day 0 to confirm that all input virus had been removed. HIV infection of NHBE cells peaks on day 3 and hence basolateral media was collected on day 3 for p24 analysis. As seen in Fig. 1, CS enhances p24 of NHBE cells by the R5-tropic BaL strain (Fig. 1a) but does not affect infection by the X4-tropic IIIB strain (Fig. 1b). To test if increased p24 expression is due to an increased viral entry, we exposed NHBE ALI cultures to CS and infected NHBE ALI cultures with the RGH-WT HIV reporter reported by Dahabieh *et al*.^20^ (and used by us^5^). RGH virus is an env^-^ single cycle HIV reporter. RGH-WT was enveloped with R5-tropic env (pBaL.01) as reported by us earlier^5^. On day 3 post-infection, total DNA was isolated from these cells and HIV DNA was quantitated by qPCR. As seen in Fig. 1c, increased levels of proviral DNA were observed in NHBE ALI cultures exposed to CS. These data demonstrate that CS enhances viral entry in NHBE ALI cultures.

### CS upregulates HIV receptors

**Figure 2:**
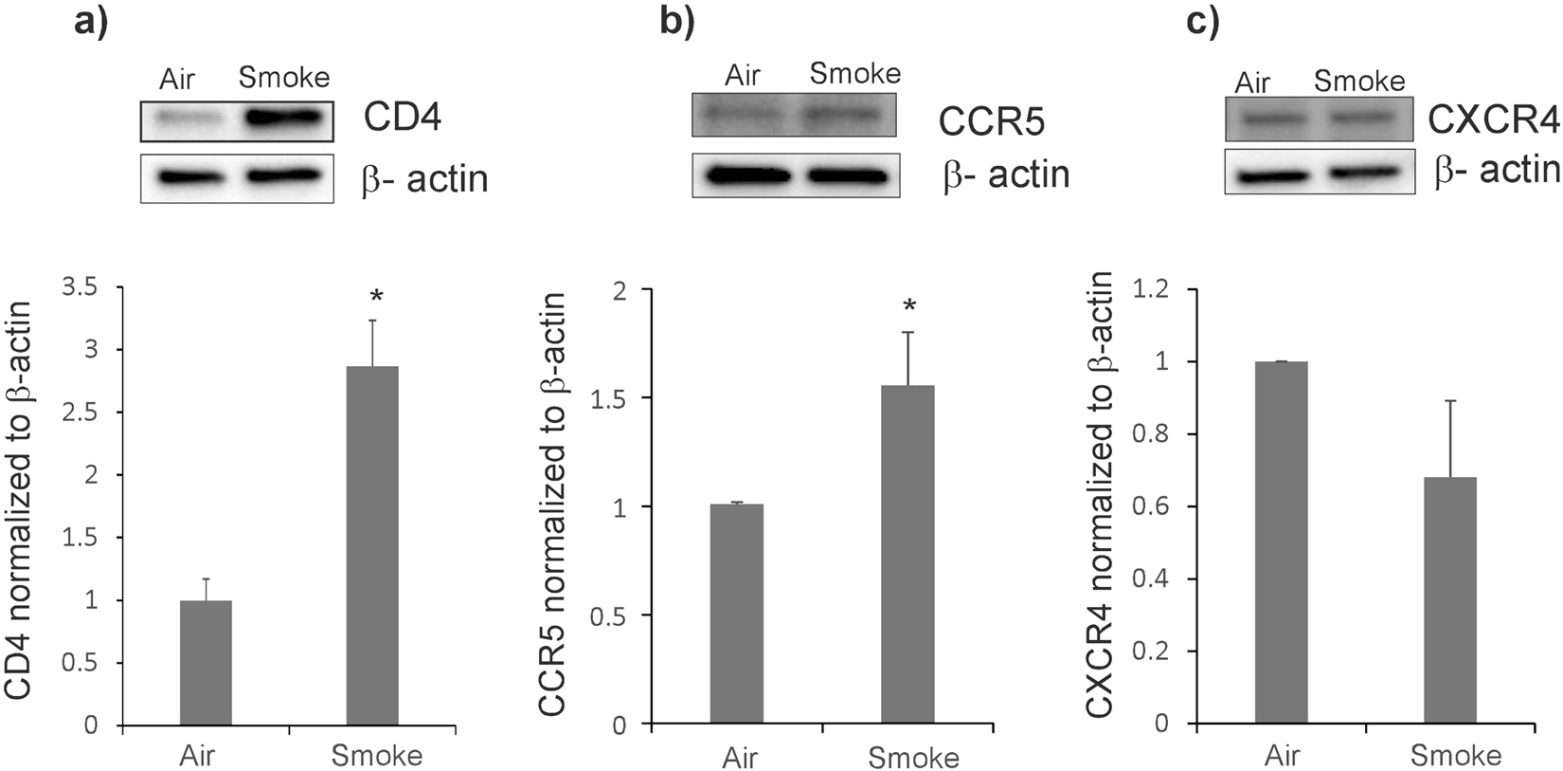
CS increases expression of HIV receptors CD4 and CCR5 in NHBE ALI cultures. NHBE ALI cultures were exposed to CS and total protein was extracted. Cells were lysed with RIPA buffer containing protease inhibitor cocktail and protein expression was quantified by Western blot analysis and normalized using β-actin. CS significantly enhances expression of CD4 (panel a) and CCR5 protein expression (panel b) when compared to air-exposed controls. CS does not increase CXCR4 protein expression (panel c). Western blot images (**a**–**c**) are representative of NHBE ALI cultures from three independent lungs. Relative density of the detected protein band was measured by using the ImageJ software and the values obtained were averaged. n = 3 lungs (unless stated otherwise). *Significant (p < 0.05).

Given reports in literature that both, CS and TGF-β (induced by CS) can upregulate HIV receptors^12–14^ in different cell types, we tried to determine if CS also affects expression of these receptors in NHBE ALI cultures. NHBE cultures redifferentiated at the ALI were exposed to CS and the expression of canonical HIV receptors CD4, CCR5 and CXCR4 were determined by western blot analysis. As seen in Fig. 2, CS significantly increases expression of the primary HIV receptor CD4 (Fig. 2a). In agreement with reports that have demonstrated that CS enhances CCR5 expression^12,13^, we also noticed a significant increase in CCR5 expression in NHBE ALI cultures exposed to CS (Fig. 2b). However, CS does not enhance expression of CXCR4 (Fig. 2c).

**Figure 3:**
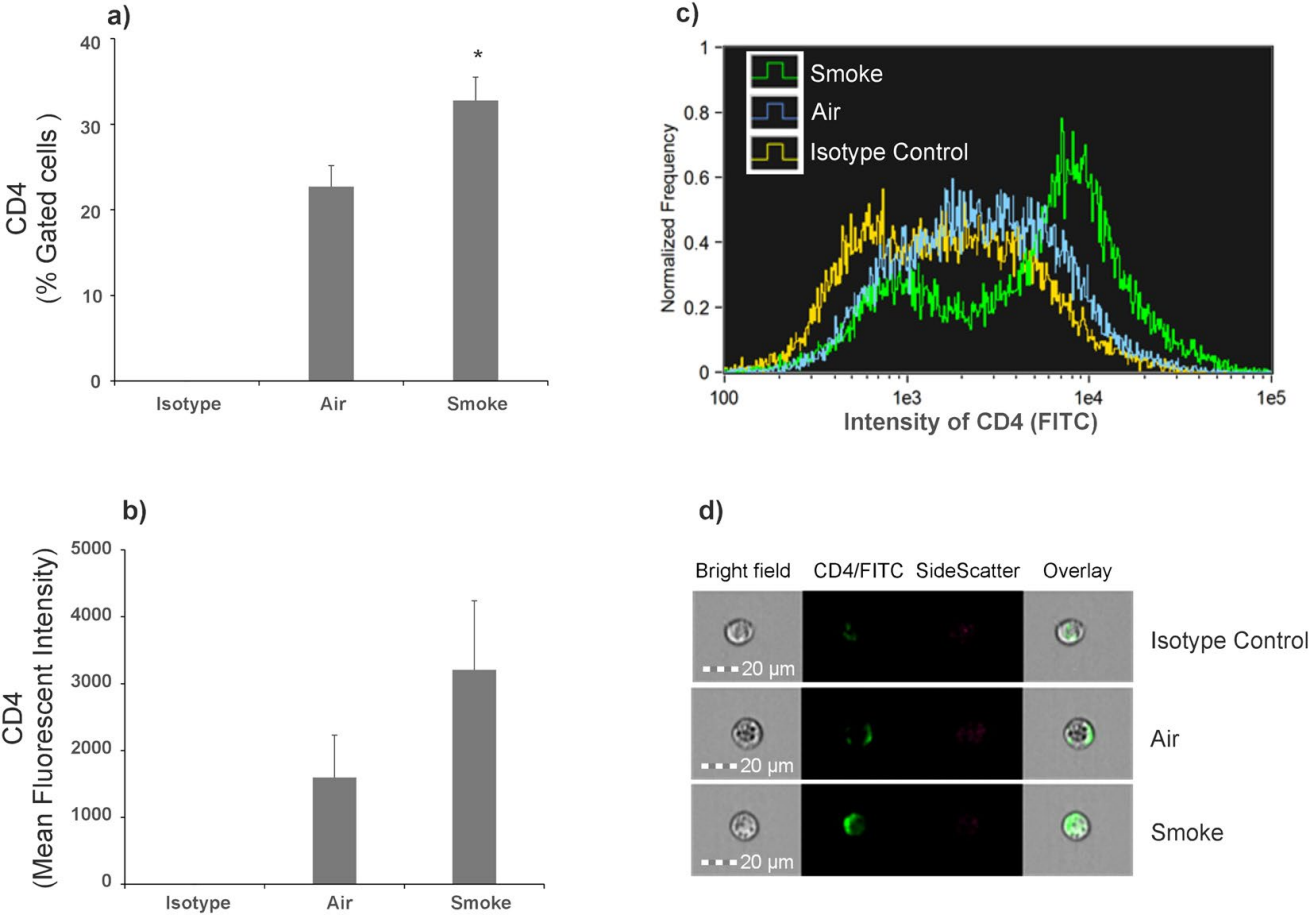
Effect of CS exposure on CD4 Cell surface receptor quantified by single cell imaging flow cytometry. NHBE ALI cultures were exposed to CS. At the end of smoking, cells were immuno-stained with surface markers anti-Human CD4 labeled with FITC. % Gated CD4+ or FITC+ cells for air NHBE (22.71 ± 2.44%) and smoke NHBE (32.78 ± 2.71%) are represented (panel a). MFI of FITC from in total population of cell images acquired (10,000 per sample) for air NHBE cells (1597.65 ± 633.43) and smoked NHBE cells (3208.24 ± 1030.86) are represented (panel b). A representative overlay histogram of intensity of FITC for isotype control (yellow), air (blue) and smoke (green) (panel c). A representative single cell image for each sample (panel d). Experiments were carried out from at least 3 different lungs. *Significant (p < 0.05).

To determine if the increase in expression relates to an increase in cell surface receptor levels, NHBE ALI cultures were exposed to CS as described in Fig. 2. Following an additional 24 hours, cell surface CD4 levels were determined by single cell imaging flow cytometry. Cells were immuno-stained with surface markers using anti-human CD4 (BD Biosciences, Cat # 555346,) labeled with FITC. Isotype controls (BD Biosciences, Cat # 555748) were also analyzed to account for nonspecific staining. Data were acquired and analyzed with Amnis® FlowSight® Imaging Flow Cytometer and IDEAS software. Staining, image collection, and analysis were carried out according to manufacturer’s protocol and as previously published^21,22^. As seen in Fig. 3, panels a-d, smoked NHBE cells showed a significantly increased percentage of CD4+/FITC+ cells (32.78 ± 2.71) compared to air NHBE cells (panel a). Mean fluorescent intensity (MFI) of FITC in the total population of cell images acquired (10,000 per sample) shows that concurrent with % gated cells, smoked NHBE cells show increased MFI compared to air NHBE cells (panel b). A representative overlay histogram of intensity of FITC for isotype control (yellow), air (blue) and smoke (green) shows a rightward shift in intensity of smoked cells indicate an increase in CD4 intensity in smoked NHBE cells compared to air-exposed NHBE cells (panel c). The representative single cell images for each sample clearly shows an increased surface expression of CD4 in smoke exposed cells compared to air-exposed controls (panel d).

**Figure 4:**
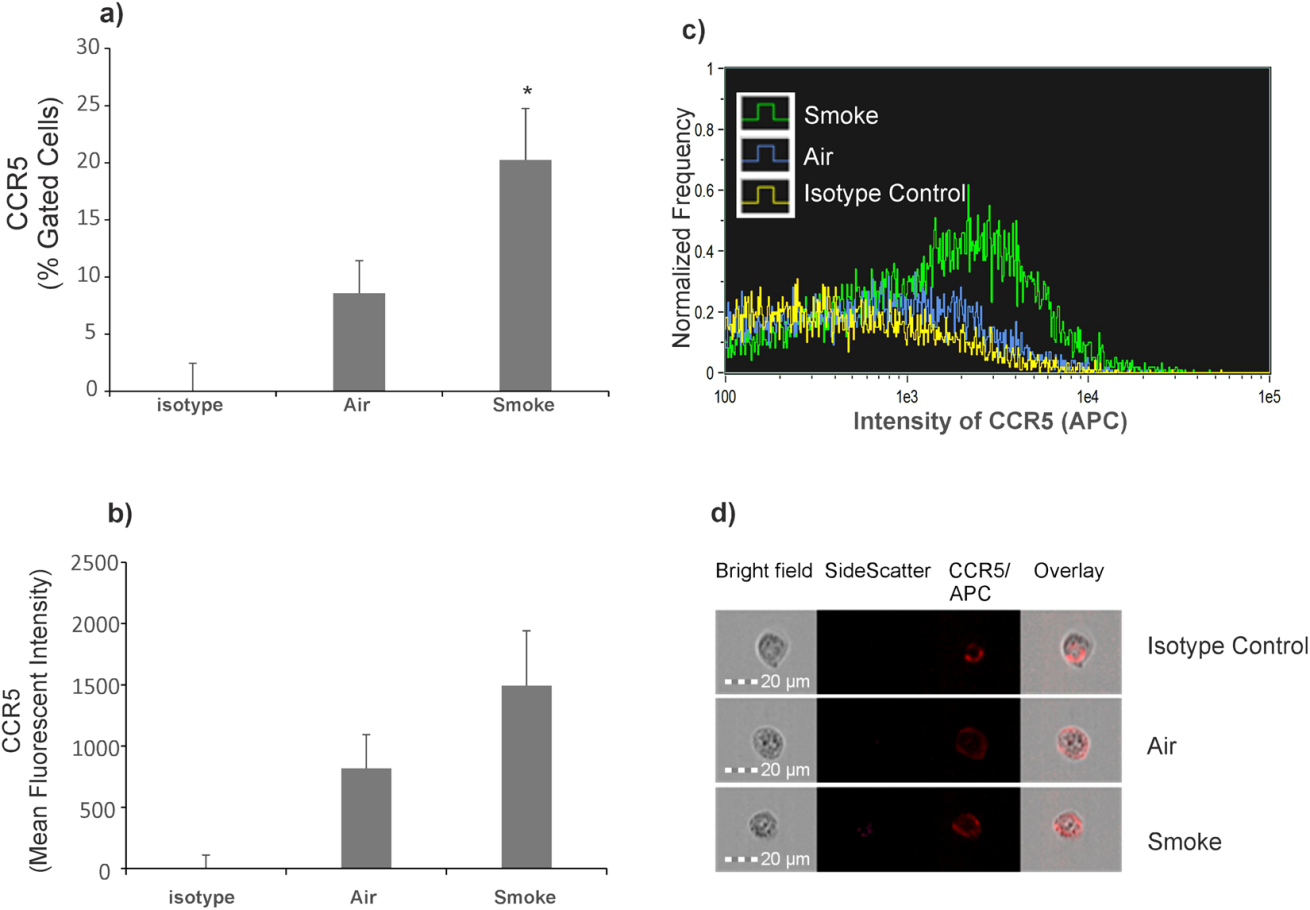
Effect of CS exposure on CCR5 Cell surface receptor quantified by Single Cell Imaging flow cytometry. NHBE ALI cultures were exposed to CS. At the end of smoking, cells were immuno-stained with surface markers anti-human CCR5 labeled with APC. Percent Gated CCR5+ or APC+ cells for air NHBE (8.58 ± 2.85%) and smoke NHBE (20.25 ± 4.5%, P = 0.03) are represented (panel a). MFI of APC from in total population of cell images acquired (10,000 per sample) for air NHBE cells (818.74 ± 274.02) and smoked NHBE cells (1494.16 ± 447.95) are represented (panel b). Representative overlay histogram of intensity of APC for isotype control (yellow), air (blue) and smoke (green) (panel c). A representative single cell image for each sample is shown (panel d). Experiments were carried out from at least 3 different lungs. *Significant (p < 0.05).

To determine if increased expression of CCR5 correlates with increased cell surface expression of CCR5, NHBE ALI cultures exposed to CS were immuno-stained with surface markers using anti-human CCR5 (BD Biosciences, Cat # 561748,) labeled with APC. Isotype controls (BD Biosciences, Cat # 555576,) were also analyzed to account for non-specific staining. As seen in Fig. 4, panels a–d, NHBE ALI cultures exposed to CS showed a significantly increased percentage of CCR5+ /APC+ cells (20.25 ± 4.5%, P = 0.03) compared to air-exposed NHBE ALI cultures (8.58 ± 2.85%) (panel a). MFI of APC in the total population of cell images acquired (10,000 per sample) shows that concurrent with % gated cells, smoked NHBE cells show increased MFI compared to air NHBE cells (panel b). A representative overlay histogram of intensity of APC for isotype control (yellow), air (blue) and smoke (green) shows a rightward shift in intensity of smoked cells indicate an increase in CCR5 intensity in smoked NHBE cells compared to air NHBE cells (panel c). The representative single cell images for each sample clearly shows an increased surface expression of CCR5 in smoke exposed cells compared to air-exposed controls (panel d). We did observe an increased background staining with our isotype controls for both CD4 and CCR5. However, in both cases, staining was intracellular suggesting a non-specific uptake mechanism of the IgG antibody possibly due to presence of Fc receptors on NHBE cells. Bronchial epithelium of human, non-human primate, and mouse express Fc receptors that are involved in Fc receptor-dependent transcytosis of IgG across mucosal barriers^23^.

### CS increases CCR5 expression by TGF-β mediated suppression of miR-141-5p

**Figure 5:**
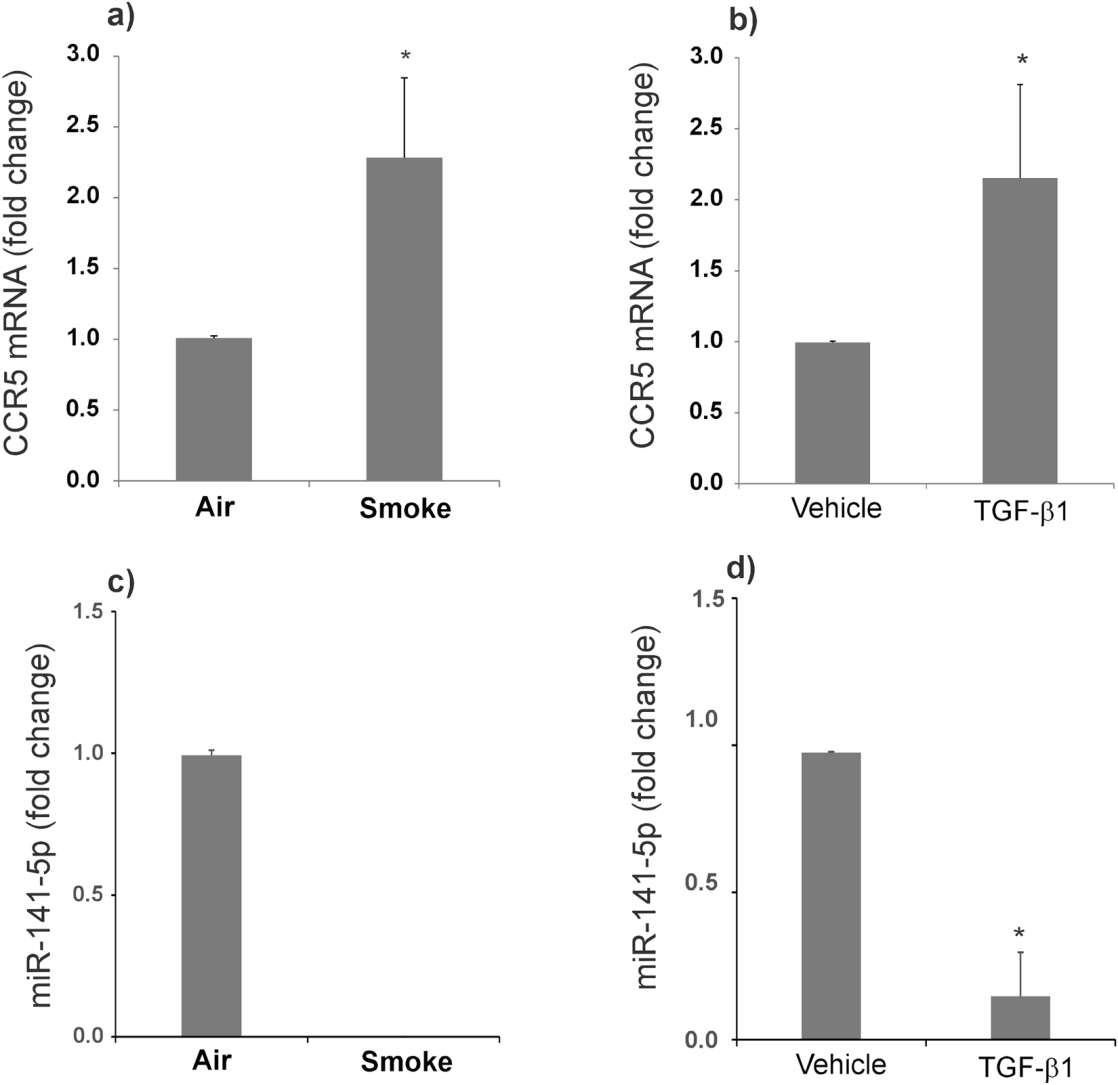
CS increases CCR5 expression by TGF-β mediated suppression of miR-141-5p. NHBE ALI cultures were exposed to CS. Total RNA was extracted and analyzed to quantify CCR5 mRNA expression by qRT-PCR. CS enhances CCR5 mRNA levels (panel a). Lung and age-matched NHBE ALI cultures were treated with recombinant TGF-β1(10 ng/ml apically and basolaterally). After 20 hours, cells were lysed and total RNA was extracted and analyzed for CCR5 mRNA expression by qRT-PCR. TGF-β1 increases CCR5 expression comparable to that observed in CS exposed cells (panel b). Total RNA was analyzed for expression of miR-141-5p from CS exposed and TGF-β treated cells by qRT-PCR. We found the miR-141-5p expression is suppressed by CS as well as TGF-β1 treatment (panels c,d). n = 3 different lungs. *Significant (p < 0.05).

A number of reports including ours have shown that CS induces TGF-β signaling^11,24,25^. TGF-β signaling has also been shown to directly suppress several microRNAs including miR-141-5p^26^, the only experimentally validated miRNA known to tightly regulate CCR5 expression via ribosomal frameshifting^27^. Primarily, we have investigated if increase in CCR5 protein levels is due to an overall increase in CCR5 mRNA. NHBE ALI cultures were exposed to CS (air as control). Total RNA was isolated and analyzed for CCR5 mRNA levels as described by us earlier^5^. As seen in Fig. 5a, CS increases CCR5 mRNA levels suggesting that increase in CCR5 by CS is due to increase in CCR5 transcripts. Given that CS induces TGF-β signaling, we tried to determine if TGF-β treatment leads to a similar increase in CCR5 mRNA levels. NHBE ALI cultures were treated with TGF-β (vehicle as control) as described by us earlier^11^. As seen in Fig. 5b, TGF-β treatment also increases CCR5 mRNA levels. Given that TGF-β suppresses miR-141-5p in other cell types, we tried to determine if TGF-β and CS also suppress miR-141-5p. We analyzed the total RNA for miR-141-5p expression using miR-141-5p specific probe (Life Technologies, Cat # 478712_mir). Figure 5(c,d) shows that both, CS and TGF-β suppress miR-141-5p suggesting that CCR5 induction by CS is via TGF-β-mediated suppression of miR-141-5p.

### CS and HIV individually and additively suppress CFTR biogenesis and function

**Figure 6:**
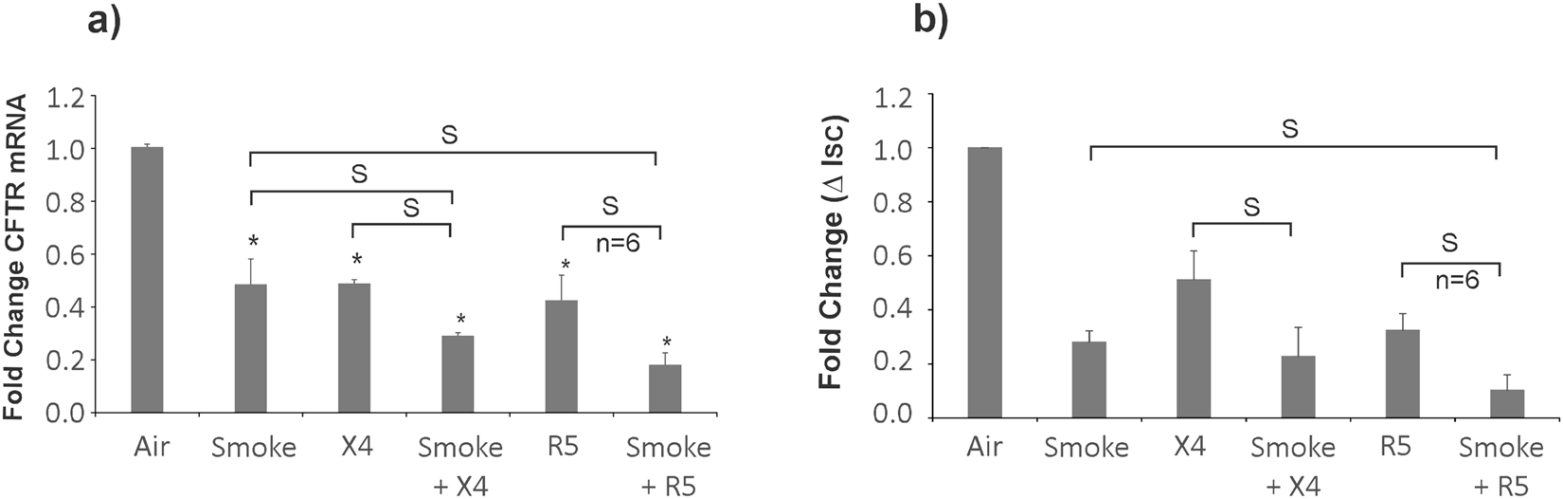
CS and HIV individually and additively suppress CFTR biogenesis and function. NHBE ALI cultures were infected with either R5- (HIV BaL) or X4- (HIV IIIB) tropic strain of HIV and exposed to chronic smoke exposure on days 0, 3, 6 and 9 (air as control). On day 9, total RNA was analyzed for CFTR mRNA by qRT-PCR. Another set of age and lung-matched cultures grown on snapwells were treated similarly and mounted in Ussing chambers on day 9. Cells were mounted in Ussing chambers and Cl^-^ efflux in response to albuterol addition was determined in the presence of amiloride as reported by us earlier^5^. HIV infection and CS individually and additively suppresses CFTR mRNA and function (panels a,b). n = 3 lungs (unless stated otherwise) *significant; S = significant from each other (p < 0.05).

Given that CS enhances HIV infection of NHBE cells (Fig. 1) and HIV Tat and CS suppress CFTR mRNA (and consequently function) via a common pathway involving TGF-β signaling^5,11^, and CS directly suppresses CFTR function by localizing them in aggregosomes^28^, we tried to determine if HIV infection and CS exposure leads to a synergistic suppression of CFTR biogenesis and function. We decided on the long-term smoke and HIV exposure to recapitulate the infection process and HIV Tat expression *in vivo*. This is because our observed kinetics with HIV infection of NHBE ALI cultures demonstrates an initial burst of viral output which gradually decreases and achieves steady state p24 production by day 9 and continues up to day 50^5^. NHBE ALI cultures were exposed to CS alone as described earlier or CS followed by HIV infection of NHBE ALI cultures. For these studies, we used a lower HIV dose of 2.5 ng/ml p24 equivalent to prevent disruption of the pseudostratified epithelial layer by HIV. Smoke exposed cells (air exposed as control) were infected with either R5 (HIV BaL) or X4 (HIV IIIB) tropic strain of HIV and exposed to chronic smoke exposure on days 0, 3, 6 and 9 post-infection (air as control). Total RNA was extracted and analyzed for CFTR mRNA levels by qRT-PCR. Exposure to CS alone or infection with either R5- or X4-tropic HIV by itself suppressed CFTR mRNA (Fig. 6a) and function (Fig. 6b). However, CS exposure exacerbated CFTR mRNA suppression in NHBE ALI cultures also infected with HIV. Another set of lung-matched NHBE ALI cultures grown on snap wells were treated identically and mounted in Ussing chambers. CFTR activation in response to β_2_-agonist albuterol was determined as described by us earlier^5,11^.

## Discussion

The airway epithelium comprises a primary barrier to occlude inhaled particles such as allergens, toxicants (CS) and pathogens. Exposure to CS promotes various pathophysiology mechanisms like oxidative stress and activation of CYP enzymes^15,16^ suppression of CFTR biogenesis^11,29^, ciliophagy and consequently MCC in NHBE cells^11,30^. Several reports have demonstrated an increased susceptibility to lung comorbidities like pneumonia, chronic bronchitis, asthma and COPD in cART era HIV population compared to non-infected age-matched controls^31,32^. These effects are exacerbated in smokers and upon cART cessation providing a clear pointer to the role of HIV replication and smoking in these lung diseases. Rao *et* *al*. have already demonstrated that oxidative stress due to CS enhances HIV replication^15^. To date no studies have addressed the interplay between CS and HIV infection in airway epithelial cells. In this manuscript, we demonstrate that CS also enhances viral entry by upregulating HIV co-receptor CCR5. We observed that CS only enhances infection of R5-tropic strain of HIV but not X4-tropic strain in age and lung-matched NHBE ALI cultures. This indicates that smoke does not affect stages downstream of viral reverse transcription. Moreover, when NHBE ALI cultures are infected with the single cycle HIV RGH virus enveloped with R5-tropic envelope, increased levels of proviral DNA are detected in smoke exposed cells suggesting that the increase in HIV replication is due to enhanced entry by HIV. Given that the NHBE ALI cultures are age- and lung-matched, and increased HIV p24 and RNA is only observed in R5-tropic HIV infected cells but not X4-tropic HIV infected cells, also rules out the possibility that increase in viral entry is due to changes in non-canonical receptors.

Since the only difference in the life cycle of the two strains is co-receptor usage we tried to determine if CS increases expression of HIV receptors. We found that while CS increases expressions of CD4 and CCR5, it does not alter expression of CXCR4. Our data also shows that CS mediated increases in protein levels correlate with flow cytometry data of cell surface CCR5 and CD4. Our data also suggests that co-receptor expression on NHBE cells is the limiting factor determining HIV infection. Indeed, NHBE ALI cultures and bronchial brushings from HIV patients expressed very high levels of CD4 (7-fold compared to CFTR) but lower levels of co-receptor CXCR4 and CCR5^5^. Since CS enhances TGF-β signaling (we have already shown effects of smoke on CFTR are mediated by TGF-β), we tried to determine if TGF-β plays a role in CCR5 upregulation. We found that TGF-β also upregulates CCR5 expression. Given reports that TGF-β suppresses several microRNAs including miR-141-5p^26^, the only experimentally validated miRNA known to regulate CCR5, we tried to determine if TGF-β and by extension CS also suppresses miR-141-5p in NHBE ALI cultures. miRNAs are endogenous non-coding RNAs of ~22 nucleotides length which mediate their regulatory effects by binding the 3′-UTR binding of the target mRNAs, causing either cleavage or translational repression based on sequence complementarity. Our data validate that CS and TGF-β both suppress miR-141-5p suggesting that CS upregulates CCR5 via the TGF-β/miR-141-5p axis. Hence exogenous delivery for miR-141-5p can serve as an important therapeutic lead to suppress CCR5 expression and protect the bronchial epithelium from infection by R5-tropic HIV.

**Figure 7:**
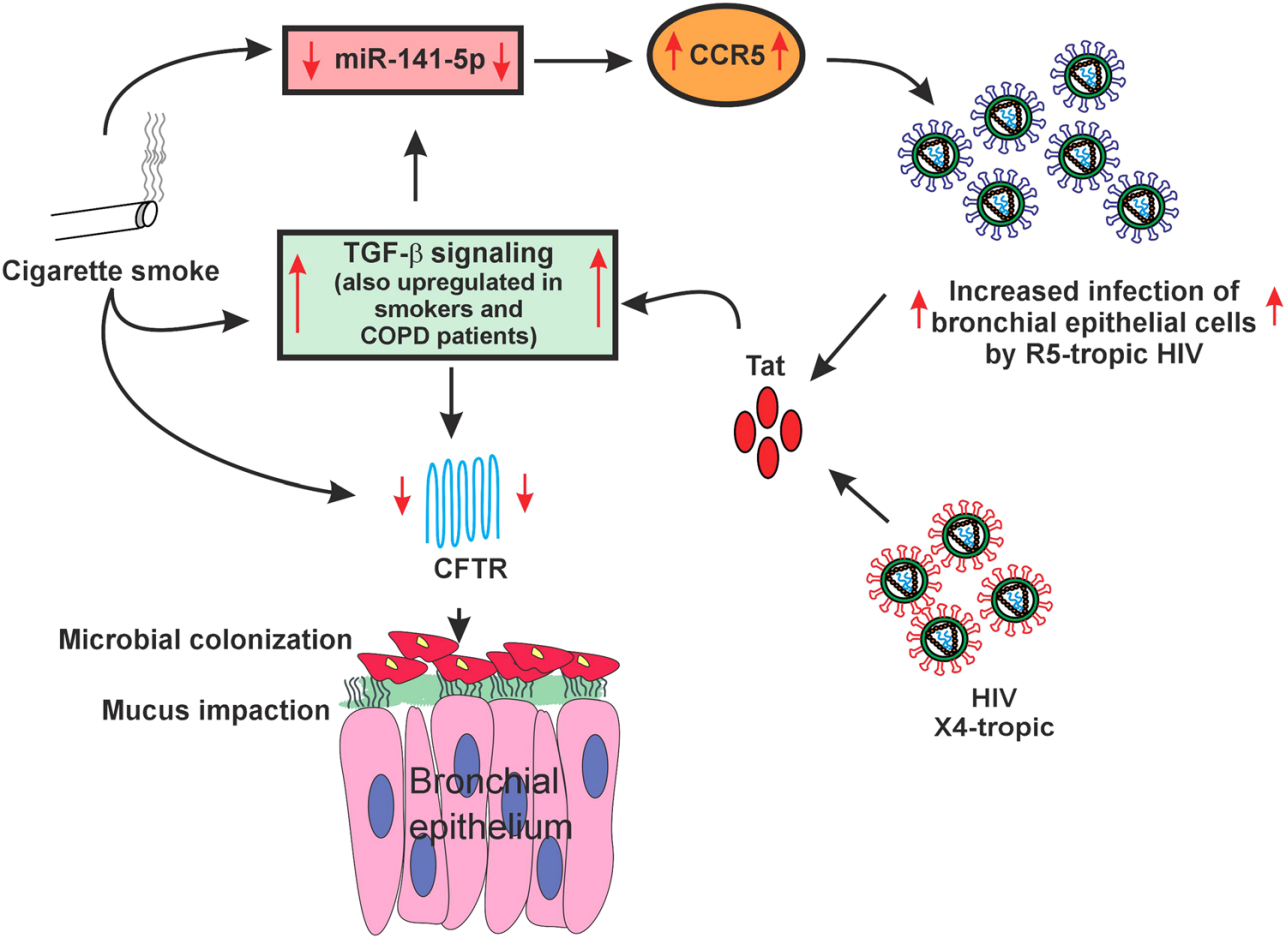
Schematic representation of interplay between CS and HIV infection to explain MCC suppression in smokers. CS suppresses miR-141-5p which in turn enhances HIV infection by R5-tropic virus. HIV Tat and CS induce TGF-β signaling and this results in additive suppression of CFTR mRNA and function. CFTR suppression consequently leads to MCC dysfunction, mucus impaction and microbial colonization.

Finally, we tried to determine if the increased interplay between CS and HIV exacerbates CFTR given that CS and HIV Tat suppress CFTR via a common pathway involving TGF-β signaling^5,11^. We found that both R5-tropic and X4-tropic strains of HIV and CS individually and additively suppress CFTR mRNA and consequently its function. This could be because CS enhances viral replication^15^, thereby increasing the Tat burden. At the same time CS and Tat also activate TGF-β signaling. We observed maximal CFTR suppression in CS exposed NHBE ALI cultures infected with R5-tropic strain possibly because CS also enhances CCR5 expression thereby increasing viral entry. Hence CS can exacerbate the effects of HIV infection in the airway with a greater exacerbation observed with R5-tropic strain of HIV. Figure 7 demonstrates a schematic representation of the interplay between CS and HIV infection whereby CS enhances viral replication and entry (at least in case of R5-tropic virus). CS and Tat suppresses miR-141-5p with a concomitant upregulation of CCR5 co-receptor. Increased infection and replication will increase the HIV Tat burden in the airway that, with CS additively suppresses CFTR biogenesis and function. While cell to cell transmission is also responsible for HIV infection, due to limitations in the culture conditions for NHBE ALI cultures (ALI medium does not allow immune cell growth^5^) we were unable to test effects of CS on cell to cell transmission of HIV. However, we believe that this would not alter our observations since cell to cell transmission also requires fusion by viral gp120 on infected cells with co-receptors on target cells^33^. Also cell free virus has been used as proxy for infection studies with NHBE cultures^5,34^.

In conclusion, our findings suggest that HIV smokers may demonstrate increased CFTR dysfunction compared to their non-smoking counterparts. Given that CFTR plays a pivotal role in MCC and several other aspects of airway innate immunity, increased CFTR dysfunction in HIV smokers can explain the increased incidence of lung infections in HIV smokers compared to HIV non-smokers.

## Materials and Methods

Primary human bronchial epithelial cells were obtained as described by Fulcher and Randall^35,36^ and re-differentiated at the ALI as reported by us^11,37^. Donor consent was obtained for research if lungs were found unsuitable for transplantation for reasons unrelated to airway disease and provided by the University of Miami Life Alliance Organ Recovery Agency (LAORA). Since the material is obtained from deceased individuals with minor, de-identified information, its use does not constitute human subjects research as defined by CFR 46.102. LAORA maintains a signed consent of each individual (or legal healthcare proxy) for donation of the lungs for research. Experiments used cells from non-smokers to not confound the findings. The primary cultures undergo differentiation at the ALI reproducing the *in vivo* morphology and key physiologic processes to regenerate the native bronchial epithelium *ex vivo*^35,36^.

### CS exposure

NHBE ALI cultures were exposed to CS using a SCIREQ smoke robot. Four cigarettes were smoked with a puff volume of 35 ml for 2 seconds every 60 seconds and blown over cell culture filter at rate of 5 ml/min according to ISO 3308. The total number of puffs was 32 for a duration of approximately 35 minutes. Smoke exposures were done 24 hours prior to infection with R5 or X4-tropic HIV. For chronic smoke exposure, cells were exposed to CS every 3 days using the same regimen.

### Virus strains and infection studies

The X4-tropic viral strain HIV IIIB was a kind gift from Dr. John Rossi (City of Hope Medical Center, Duarte, CA) and R5-tropic viral strain HIV BaL was a kind gift from Dr. Madhavan Nair (Department of Immunology, Institute of Neuroimmune Pharmacology, Herbert Wertheim College of Medicine, Florida International University. Miami, FL). NHBE ALI cultures grown on transwells were infected apically and basolaterally with of either X4-tropic HIV strain (IIIB) or R5-tropic HIV strain (BaL) (5 ng/ml p24 equivalent) as described earlier^5^. 16 hours post infection, cells were washed apically and basolaterally with PBS four times to remove any residual input virus. The fourth wash was collected for p24 analysis and measured as day 0 to confirm that all input virus had been removed. Culture supernatants were collected on day 3 and p24 antigen levels were determined using p24 ELISA kit (ZeptoMetrix Corp. Cat # 0801200) according to manufacturer’s instructions. For chronic HIV exposure, NHBE ALI cultures grown in snap wells cells were infected with X4-tropic HIV strain (IIIB) or R5-tropic HIV strain (BaL) (2.5 ng/ml p24 equivalent). 16 hours post-infection, cells were washed apically and basolaterally with PBS four times to remove any residual input virus. Infection was allowed to proceed for 9 days. For single cycle HIV infection, RGH virus was enveloped with R5-tropic envelope as reported by us earlier^5^. NHBE ALI cultures were infected with R5-tropic RGH virus. 16 hours post-infection, cells were washed apically and basolaterally with PBS four times to remove any residual input virus. 3 days post-infection, total DNA was isolated and quantitated for HIV LTR as reported in our earlier manuscript. Copy numbers were determined using comparative Ct method with known copies of HIV DNA.

### Electrophysiology experiments

CFTR activation was determined by Ussing chamber methods as reported by us earlier^11^. The initial resistance of each filter was measured by application of 1 mV bipolar pulses of 2 seconds’ duration. Amiloride (10 µM) was added apically to inhibit epithelial sodium channel (ENaC) influences. To measure CFTR, albuterol was added and short circuit current (ΔIsc) was allowed to stabilize. ΔI_SC_ in response to albuterol was determined as an index of CFTR function.

### Quantitative RT-PCR

For qRT-PCR analysis total RNA was extracted using the Qiagen RNeasy mini kit (Cat # 74104) and complementary DNA (cDNA) was reverse transcribed using the Applied Biosystems high-capacity cDNA reverse transcription kit (Cat # 4368814). qRT-PCR was performed on the Bio-Rad CFX96 real-time system using validated TaqMan probes (Life Technologies/Applied Biosystems CD4, Cat # HS01058407-m1; CXCR4, Cat # HS00607978-s1;CCR5, Cat # HS99999149-s1; CFTR, Cat # HS00357011-m1 and GAPDH, Cat # Hs02758991_g1). qRT-PCR results are represented as relative quantification normalized against internal control (GAPDH).

### miR-141-5p qPCR experimental protocol

NHBE ALI cultures were exposed to CS (air as control) or TGF-β (vehicle as control). Following 24 hours total RNA was extracted from exposed to CS and TGF-β treated with NHBE ALI cultures by using the Qiagen RNeasy mini kit (Cat # 74104) and cDNA was reverse transcribed by the Applied Biosystems TaqMan™ Advanced miRNA cDNA Synthesis Kit (Life Technologies/Applied Biosystems, Cat # A28007) according to the manufacturer’s instructions. Real time qPCR was done using TaqMan™ Fast Advanced Master Mix (Life Technologies/Applied Biosystems, Cat # 4444557) in combination with validated TaqMan probes (Life Technologies/Applied Biosystems, hsa-miR-141-5p, Cat # 478712_mir) according to the manufacturer’s directions. qRT-PCR results are represented as relative quantification normalized against internal control (GAPDH).

### Western Blot analysis

Total protein was loaded onto a gel and run at 100 V. Protein was transferred to PVDF membrane. Following blocking in 5% milk primary antibodies; CD4 (1: 500; Sigma-Aldrich, Cat # HPA004252), CXCR4 (1:1000; ThermoFisher scientific, Cat # PA5-19857), CCR5 (1:1000; ThermoFisher scientific, Cat # PA1-41303) and β- actin (1:1000; Cell Signaling, Cat # 4970) were added. Blot was incubated in an anti-rabbit secondary antibody diluted to a concentration of 1:2500. Bands were detected in Chemidoc (Bio-Rad Laboratories, USA) using supersignal west femto maximum sensitivity substrate (ThermoFisher scientific, Cat # 34095) in accordance with the manufacturer’s instructions. Quantitative densitometry analyses were performed using the Quantity One software (Bio-Rad Laboratories, USA) and the density values are normalized to β-actin.

### Flow Cytometry analysis

NHBE ALI cultures were exposed to CS. At the end of smoking, cells were immuno-stained with surface markers anti-Human CD4 (BD Biosciences, Cat # 555346,) labeled with FITC, anti-human CCR5 (BD Biosciences, Cat # 561748) labelled with APC. Isotype controls (BD Biosciences, Cat # 555748, Cat # 555576,) were also analyzed to account for nonspecific staining. Data were acquired with Amnis^®^ FlowSight^®^ Imaging Flow Cytometer. Staining and image collection were carried out according to manufacturer’s protocol and as previously published^22^. Images for compensation were collected with compensation beads (552843, BD Biosciences) labeled with the same antibody.

### Statistical analysis

Unless otherwise mentioned, data were expressed as mean ± SEM from NHBE ALI cultures from at least three lungs. The data were subjected to statistical analysis using unpaired t-tests for two groups or ANOVA followed by Tukey Kramer honestly significant difference test for multiple comparisons as appropriate. The significance was considered at the level of P < 0.05.

## Electronic supplementary material

Supplementary Data
