# Supplementary material for: Cigarette smoke promotes HIV infection of primary bronchial epithelium and additively suppresses CFTR function
Source: Sci Rep. 2018 May 22;8:7984. doi: 10.1038/s41598-018-26095-z (PMC5964097; doi:10.1038/s41598-018-26095-z)
Supplement: Supplementary file 1 — Supplementary Data [file 41598_2018_26095_MOESM1_ESM.pdf]

Cigarette smoke promotes HIV infection of primary bronchial epithelium and additively suppresses CFTR function.

Chinnapaiyan S[1]., Dutta R[1]., Bala J[1]., Parira T[1]., Agudelo M[1]., Nair M[1]., and Unwalla HJ[1] *.

* To whom correspondence should be addressed.

Hoshang Unwalla, PhD, Florida International University, Department of Immunology, Institute of Neuroimmune Pharmacology, 11200 SW 8th street NW 10th Ave., AHC-1 # 421, Miami, FL 33199. Tel.: 305-348-3442; Fax: 305-243-6992; email: hunwalla@fiu.edu

1. Department of Immunology, Institute of Neuroimmune Pharmacology, Herbert Wertheim College of Medicine, Florida International University. Miami, FL

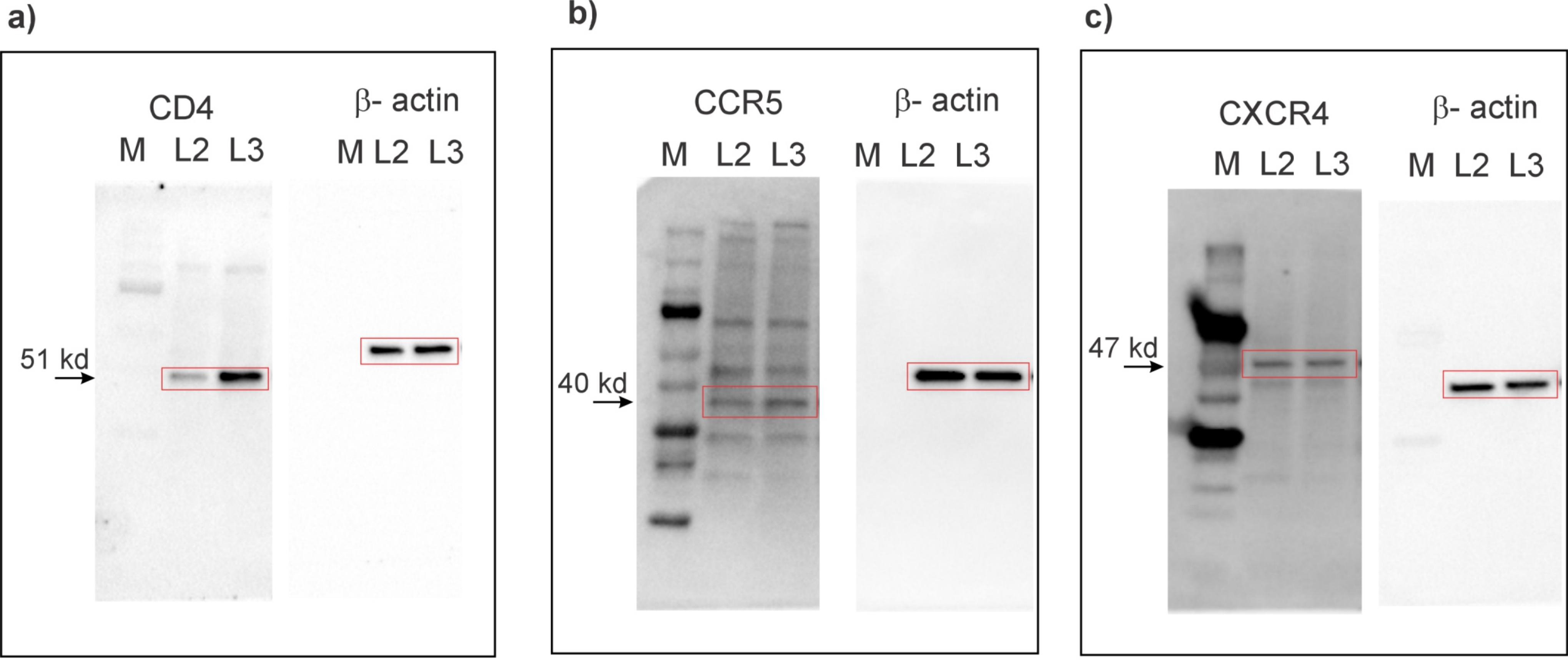

Full length western blot images. Panel a) CD4; Panel b) CCR5, Panel c) CXCR4.
M: Protein marker; Lane 2: Air; Lane 3: Smoke; Red color highlighted bands were used for our manuscript.

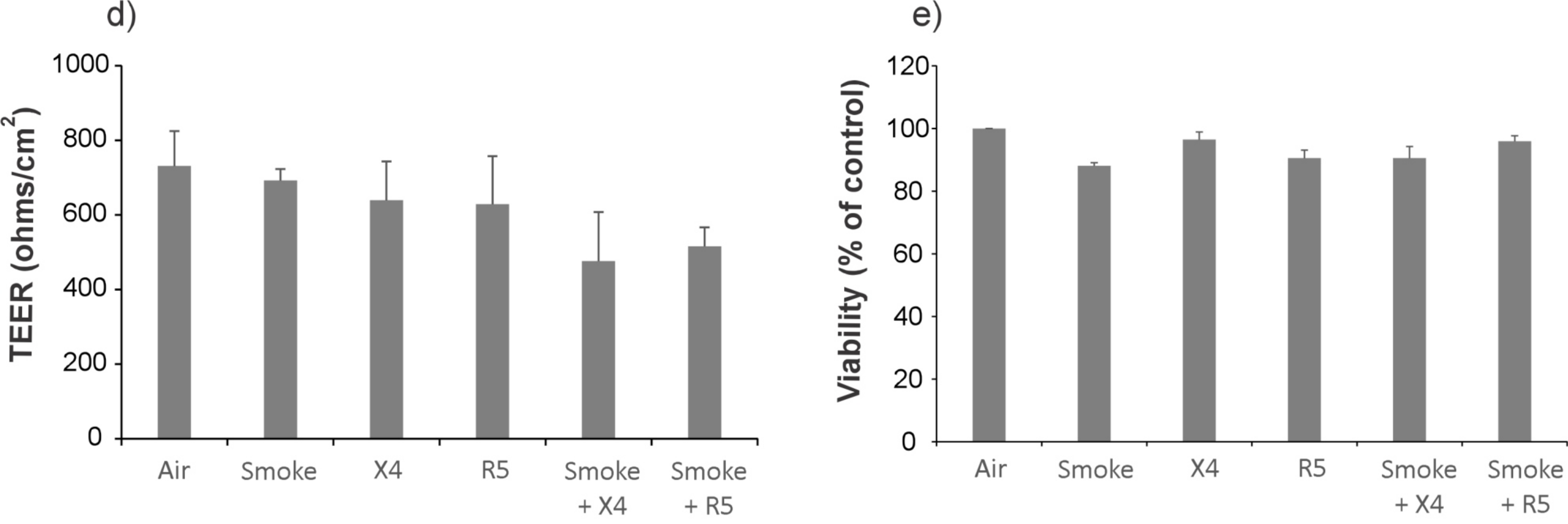

Panel d: Transepithelial electrical resistance (TEER) was measured following chronic exposure to HIV and/or CS. NHBE ALI cultures exposed to HIV and CS did show a trend towards a decreased TEER. Panel e: Cell viability assay demonstrates no significant change in viability in HIV or smoke exposed cells from Air exposed controls with the smoke and HIV p24 dose used.
